# Relationships between self-report and cognitive measures of hearing aid outcome

**DOI:** 10.1179/205057113X13782848890774

**Published:** 2013-12

**Authors:** Elaine Hoi Ning Ng, Mary Rudner, Thomas Lunner, Jerker Rönnberg

**Affiliations:** 1 Linnaeus Centre HEAD, Department of Behavioural Sciences and Learning, Swedish Institute for Disability Research, Linköping University, Sweden; 2 Linnaeus Centre HEAD, Department of Behavioural Sciences and Learning, Swedish Institute for Disability Research, Linköping University, Sweden; Eriksholm Research Centre, Oticon A/S, Snekkersten, Denmark; and Department of Clinical and Experimental Medicine, Linköping University, Sweden

**Keywords:** Hearing aid, Outcome measures, Self-reported, Working memory, Cognitive abilities

## Abstract

This present study examined the relationship between cognitive measures and self-report hearing aid outcome. A sentence-final word identification and recall (SWIR) test was used to investigate how hearing aid use may relate to experienced explicit cognitive processing. A visually based cognitive test battery was also administered. To measure self-report hearing aid outcome, the International Outcome Inventory – Hearing Aids (IOI-HA) and the Speech, Spatial and Qualities of Hearing Scale (SSQ) were employed. Twenty-six experienced hearing aid users (mean age of 59 years) with symmetrical moderate-to-moderately severe sensorineural hearing loss were recruited. Free recall performance in the SWIR test correlated negatively with item 3 of IOI-HA, which measures residual difficulty in adverse listening situations. Cognitive abilities related to verbal information processing were correlated positively with self-reported hearing aid use and overall success. The present study showed that reported residual difficulty with hearing aid may relate to experienced explicit processing in difficult listening conditions, such that individuals with better cognitive capacity tended to report more remaining difficulty in challenging listening situations. The possibility of using cognitive measures to predict hearing aid outcome in real life should be explored in future research.

## Introduction

Speech audiometry is widely used in research and clinics to objectively evaluate different hearing aid models and settings (e.g. compression and noise reduction), as well as hearing aid fittings. However, conventional speech tests may not be sensitive in predicting hearing aid outcome in daily life (Taylor, 2007). Therefore, it is valuable for both clinicians and researchers to understand the perceived performance of hearing devices and their algorithms in real life and to determine whether the users are satisfied with the devices. This kind of information, however, cannot be assessed using behavioral tests and these must therefore be complemented with subjective self-reports about users' real-life experience. Some studies have demonstrated an association between audiological measures (such as pure-tone and speech perception thresholds) and self-assessed hearing abilities and benefits (e.g. Humes, 2003; Kochkin, 2003). Recent studies have shown connections between speech perception in noise performance and cognitive abilities in hearing aid users (e.g. Foo *et al*., 2007; Gatehouse *et al*., 2006; Humes, 2007; Lunner, 2003; Rudner *et al*., 2009, 2012). However, investigation of the relationship between cognitive measures and self-report hearing aid outcome is rare in the literature. This relationship was investigated in the present study by studying the associations between self-report on two questionnaires measuring hearing aid outcome in real life and a recall test for speech in noise as well as a visually based cognitive test battery.

Clinically, there are a number of questionnaires that quantify hearing aid outcome on different dimensions. These include the Satisfaction with Amplification in Daily Life (SADL; Cox and Alexander, 2001); the Abbreviated Profile of Hearing Aid Benefit (APHAB; Cox and Alexander, 1995); the Hearing Handicap Inventory for Adults (HHIA; Newman *et al*., 1990)/for the Elderly (HHIE; Ventry and Weinstein, 1982); the International Outcome Inventory – Hearing Aids (IOI-HA; Cox and Alexander, 2000), which is a concise questionnaire measuring seven different outcome domains (see Table 1); and the Speech, Spatial and Quality of Hearing Scale (SSQ; Gatehouse and Noble, 2004; see Table 2 for scales and subscales of SSQ). Some studies have shown that self-reported outcome is associated with conventional audiological measures. For example, Newman *et al*. (1990) showed significant correlations between self-reported hearing handicap measured using HHIA and audiometric and speech recognition thresholds. However, some other studies have shown that self-report and audiometric measures may not agree with each other. For example, a discrepancy in measured and reported benefits has been observed in situations where background noise is loud and speech understanding is difficult. Cox and Alexander (1992) showed that objective benefit of hearing aid declines but self-reported benefit increases as listening conditions became more adverse, and correlations between these two measures of benefit were significant only when the listening conditions were less adverse. The lack of correlation in more adverse conditions might have been due to the fact that the listening environments described in the questionnaires were not perceived to be the same as those represented by the tested speech-in-noise conditions. Other factors that affect speech perception and understanding, such as central auditory processing and cognitive ability, may also influence self-perceived ability to listen in a manner that cannot be predicted from objective measures (e.g. Demeester *et al*., 2012; Kramer *et al*., 2006; Newman *et al*., 1990).

**Table 1:** Means and SDs for the IOI-HA (*n* = 25)

IOI-HA items	Mean (max = 5)	SD
1. Hours of daily use		
*Think about how much you used your present hearing aid(s) over the past two weeks. On an average day, how many hours did you use the hearing aid(s)?*	4.2*	1.1
2. Benefit		
*Think about the situation where you most wanted to hear better, before you got your present hearing aid(s). Over the past two weeks, how much has the hearing aid helped in that situation?*	4.3	0.9
3. Residual activity limitations		
*Think again about the situation where you most wanted to hear better. When you use your present hearing aid(s), how much difficulty do you STILL have in that situation?*	3.0	0.8
4. Satisfaction		
*Considering everything, do you think your present hearing aid(s) is worth the trouble?*	4.5	1.0
5. Residual participation restriction		
*Over the past two weeks, with your present hearing aid(s), how much have your hearing difficulties affected the things you can do?*	3.9	1.2
6. Impact on others		
*Over the past two weeks, with your present hearing aid(s), how much do you think other people were bothered by your hearing difficulties?*	3.5	1.1
7. Quality of life		
*Considering everything, how much has your present hearing aid(s) changed your enjoyment of life?*	4.1	1.0
Global	27.5	4.8

*For item 1, mean score of 4.2 corresponds to an average daily use of approximately 4–8 hours. However, the participants reported an average of 9.5 hours a day. This discrepancy is due to that fact that the maximum score represents a daily use of more than 8 hours, and 17 out of 25 participants, who reported a daily use of 9–15 hours, rated 5 out of 5, and the rest, who reported less than or equal to 8 hours, rated 4 or below. Therefore, the mean score here does not accurately reflect the actual average daily use.

**Table 2:** Means and SDs for SSQ scales and subscales (*n* = 26).

SSQ scales	Subscales	Mean (max = 10)	SD
Speech hearing		5.4	2.7
	Speech in quiet	7.5	1.8
	Speech in noise	4.5	2.3
	Speech in speech contexts	5.6	2.6
	Multiple speech-stream processing and switching	4.3	2.8
Spatial hearing		6.1	2.5
	Localization	6.3	2.5
	Distance and movement	5.9	2.6
Qualities of hearing		6.3	2.6
	Sound quality and naturalness	7.2	2.0
	Identification of sounds and objects	7.6	2.4
	Segregation of sounds	5.9	2.5
	Listening effort	4.7	2.6
Overall		5.9	2.6

Questions regarding the ability to perceive or to understand speech in different listening conditions when listening is aided and/or unaided are common to all the aforementioned questionnaires. Cognition plays a role in both speech perception and speech understanding, particularly in adverse listening conditions (Mattys *et al*., 2012). It has been hypothesized by Rönnberg (2003) and Rönnberg *et al*. (2008) that when speech is perceived in a favorable situation, language inputs are bound together rapidly and automatically to form phonological streams of information that unlock lexical information. This processing is automatic and implicit. Implicit processing occurs when the input is intact and matches readily with the phonological representation in long-term memory corresponding to semantic and contextual information. However, in adverse listening conditions, processing becomes explicit and effortful because extra processes are engaged to match the suboptimal input with the contents of the long-term memory store. Due to the involvement of cognition in listening in adverse conditions, there may be associations between cognitive abilities related to verbal information processing, namely speed of processing, phonological–lexical processing and verbal working memory (Rönnberg *et al*., 2008), and self-assessed speech perception or speech understanding performance in difficult listening situations.

### Hearing aid use and cognitive measures

Hearing aids are designed to enhance speech intelligibility and ultimately improve communication for people with hearing impairment (Edwards, 2007). This is achieved primarily by amplifying speech sounds and reducing undesirable noise. In addition to providing improvement at the perceptual level, use of hearing aids may also have an impact on higher cognitive function (Lunner *et al*., 2009). Using hearing aids may reduce the processing load involved in speech perception in noise and thereby increase cognitive resources available for the storage and processing of heard information (Ng *et al*., 2013).

The relationship between speech recognition in noise performance in persons with hearing impairment and cognitive ability has been well established (Akeroyd, 2008). This indicates the possibility of predicting hearing aid outcome based on individual cognitive abilities. Individuals with good cognitive abilities are more likely to benefit from amplification and advanced signal processing in hearing aids (e.g. Akeroyd, 2008; Arehart *et al*., 2013; Foo *et al*., 2007; Gatehouse *et al*., 2003; Humes, 2007; Lunner, 2003; Lunner and Sundewall-Thorén, 2007; Ng *et al*., 2013; Rudner *et al*., 2011; Sarampalis *et al*., 2009). For example, Lunner (2003) found that performance on the reading span test, which is a working memory capacity measure, and the rhyme judgment test (Lyxell, 1994), which measures the quality of phonological representations and phonological processing speed, correlated positively with the signal-to-noise ratios (SNRs) required to achieve 40% speech recognition using lists of low-redundancy five-word sentences (Hagerman and Kinnefors, 1995) in both aided and unaided conditions.

### Aims

The present study examined the relationship between cognitive measures and self-report hearing aid outcome. A sentence-final word identification and recall (SWIR) test (Ng *et al*., 2013), which measures memory for audible speech heard in different types of background noise with and without the hearing aid noise reduction system, was used to quantify the cognitive outcome of hearing aid amplification with and without signal processing in different background noise conditions. A visually based cognitive test battery was also administered to measure individual cognitive abilities related to speech processing. To measure self-report hearing aid outcome, the IOI-HA and SSQ questionnaires were employed. These two questionnaires were chosen because they have been extensively used in hearing aid-related research and to measure hearing aid benefit in real life. The IOI-HA covers the major dimensions of outcome including use, benefit and handicap; and the SSQ measures hearing disabilities across several domains, namely speech perception in various contexts and specific listening situations, the ability to segregate sounds and simultaneous speech streams and ease of listening.

We predicted that the self-reported speech understanding performance in difficult listening situations would be associated with individual cognitive abilities concerning verbal information processing and also with performance on the SWIR test. Previous studies established a relationship between cognitive abilities and aided speech recognition performance, such that better speech recognition in noise is related to high working memory capacity (e.g. Lunner, 2003). In other words, in a noisy condition, additional or explicit processing is involved in perceiving speech and consequently having more cognitive resources would lead to better speech recognition (Rönnberg, 2003; Rönnberg *et al*., 2008). Better aided speech recognition in noise is found to be associated with less reported hearing handicap (Saunders and Forsline, 2006). Thus, we predicted that individuals who have good cognitive abilities would report less hearing handicap.

## Method

### Participants

The participants in this study were identical to those reported in Ng *et al*. (2013). Twenty-six native Swedish speakers (15 women and 11 men) were recruited from the audiology clinic of the University Hospital of Linköping, Sweden. Their average age was 59 years (SD = 7, range: 32 to 65 years) and they had symmetrical moderate to moderately–severe sensorineural hearing impairment (the mean pure-tone average at 0.125, 0.25, 0.5, 1, 2, 3, 4, 6 and 8 kHz was 49.8 dB HL, SD = 6.4). All were hearing aid users, with an average daily usage of 9.5 hours over 9 years, and three of them preferred to wear one instead of two aids in everyday use. They reported no previous history of otological problems or psychological disorders. The study was approved by the Regional Ethical Review Board in Linköping and informed consent was obtained from all participants.

### Assessment instruments

#### IOI-HA

The Swedish version of IOI-HA (see Brännström and Wennerström, 2010; Öberg *et al*., 2007) was used in the study. The IOI-HA questionnaire consists of seven items, which assess seven different domains of hearing aid outcome (see Table 1). Each participant responded to each item on a 5-point scale, from the poorest outcome, which is scored as 1, to the best outcome, which is scored as 5. The questions are practically oriented and easy to read, so that the questionnaire can be completed without assistance.

The IOI-HA questionnaire is a brief and general questionnaire covering major dimensions of outcome in a concise manner, so that it can be appended to other self-report measures for any research project and its results can be compared directly across studies (Cox *et al*., 2002). The seven-item IOI-HA can be further grouped into two factors. Factor 1, which deals with improvements/benefits of hearing aid and the interaction of the individual with the hearing aid, consists of items 1, 2, 4 and 7; Factor 2, which deals with hearing handicap, residual problems and the interaction between the individual and other people, includes items 3, 5 and 6 (Cox and Alexander, 2002; Kramer *et al*., 2002).

#### SSQ

SSQ, developed by Gatehouse and Noble (2004), was used to evaluate hearing abilities in everyday life. The questionnaire consists of 50 items and covers three major aspects of hearing: Speech hearing, Spatial hearing, and Qualities of hearing. The Speech scale assesses daily listening in quiet and in the presence of different competing sounds, such as environmental noise, reverberation and other voices. The Spatial scale evaluates the ability to locate sound and discriminate distance. The Qualities scale addresses issues of sound segregation, naturalness of sound, and listening effort. Gatehouse and Akeroyd (2006) further reorganized the 50 items into 10 subscales (see Table 2), which allow more precise evaluations of hearing function in different listening scenarios. All items are rated on a 10-point scale and the participants were told to rate their aided hearing abilities. The Swedish translation of the SSQ was used in the study.

#### Speech recognition test

Aided speech reception threshold (SRT) at 84% speech intelligibility was obtained using the Swedish Hearing In Noise Test (HINT) (Hällgren *et al*., 2006) with a modified adaptive up-down procedure suggested by Levitt (1971). This test was performed using an experimental hearing aid (see description of Procedure for details) with linear amplification and individually prescribed frequency response. Each participant was required to listen to and repeat three original lists of Swedish HINT sentences (i.e. 30 sentences in total) in the standard Swedish HINT noise. None of these sentences were used in the SWIR test. Both speech and noise were first presented at 65 dB A (i.e. 0 dB SNR). The presentation level of the noise varied according to the participant's response. The SNR decreased one step when four consecutive sentences were repeated correctly and increased one step whenever a sentence was not repeated correctly. The step size was 2 dB for the first 15 sentences and was then refined to 1 dB from the 16th sentence onwards. The details of the test are described in Ng *et al*. (2013).

#### SWIR test

This test examines the effects of background noise and noise reduction signal processing in hearing aids on memory for heard sentences. There were two tasks in this test. The participants had to listen to 40 lists of eight sentences at an individualized signal-to-noise ratio predicting 95% speech intelligibility to optimize speech perception. They were instructed to report the final word of each sentence immediately after listening to it (identification task). After the final word of the eighth sentence was reported, they were prompted to recall all the final words that they had previously reported (free recall task). In this paper, we report the recall performance from the five background conditions analyzed in Ng *et al*. (2013); a quiet condition and four noise conditions: two types of background noise, which are steady-state noise (SSN) and 4-talker babble (4T), in two noise reduction settings, which are without noise reduction (NoP) and with the realistic version of binary masking noise reduction algorithm (NR) (Boldt *et al*., 2008; for details of binary masking, see Wang *et al*., 2009). Therefore, the four noise conditions are SSN/NoP, SSN/NR, 4T/NoP, and 4T/NR.

#### Cognitive test battery

This battery assesses verbal information processing speed, lexical access speed, quality of phonological representations and working memory capacity. All have been found to be important for speech understanding under adverse listening conditions (Rönnberg *et al*., 2008). The tests were visually based and stimuli were shown in the center of a computer screen. The participants had to respond as accurately and quickly as possible.

##### Physical matching

The task was to judge whether the two tokens of the same letter shown on the screen were identical in physical shape (e.g. A-A, but not A-a) (Posner & Mitchell, 1967). This test measures general processing speed and is scored based on reaction time for correct trials.

##### Lexical decision making

The task was to judge whether a string of three letters shown was a real Swedish word (e.g. ‘kub’, meaning ‘cube’) or not (e.g. ‘tra’, which is meaningless in Swedish). This test measures lexical access speed and is scored based on reaction time for correct trials.

##### Rhyme judgment test

The test was to judge whether two words of equal length rhymed or not (Baddeley & Wilson, 1985). There were four experimental conditions: (1) the words rhymed and were orthographically similar (e.g. ‘fritt’-‘vitt’, meaning ‘free’-‘white’) (2) the words rhymed but were orthographically dissimilar (e.g. ‘dags’-‘lax’, meaning ‘time’-‘salmon’), (3) the words did not rhyme but were orthographically similar (e.g. ‘salt’-‘saft’, meaning ‘salt’-‘fruit-syrup’), and (4) the words did not rhyme and they were orthographically dissimilar (e.g. ‘kalk’-‘stol’, meaning ‘lime’-‘chair’). Thirty-two word pairs were presented and half of them rhymed (conditions 1 and 2). This test is a measure of the quality of the phonological representations in the lexicon (Lyxell, 1994). It is scored based on percent correct.

##### Reading span

This test consisted of two parallel tasks. First, the participants had to judge, whether three-word sentences shown at the centre of a computer screen, at a rate of 800 ms per word with an inter stimulus interval of 75 ms, were sensible or absurd (Baddeley *et al*., 1985). The sentences were presented in lists. After each list of sentences, the participants were prompted to recall either the first or the final word of all the sentences in the list in correct serial order. Lists of three, four, and five sentences were presented in ascending order of length, and two lists were presented at each list length. The test was scored by the total number of items correctly recalled irrespective of serial order. This scoring procedure was used to optimize the individual variation in response and has been adopted in other studies (e.g. Lunner, 2003; Foo *et al*., 2007). The test used in this study was a short version of the original Swedish reading span test created by Rönnberg *et al*. (1989). A total of 24 instead of 54 sentences were presented. This test measures working memory capacity and indicates the ability to process and store verbal information simultaneously.

### Procedure

The data were collected at two sessions of approximately 2 hours each as part of a larger study. Audiometric measurements, the cognitive test battery and the IOI-HA and SSQ questionnaires were administered in the first session. The participants were instructed to fill out the questionnaires based on their experience with their own hearing aids. The objective measures, which are the HINT and SWIR tests, were performed in the second session.

Both the HINT and the SWIR were administered in a double-walled sound booth. The auditory stimuli were presented by a computer, amplified through an Oticon Epoq XW behind-the-ear hearing aid in a Brüel & Kjær anechoic test box (type 4232) equipped with an IEC 711 ear simulator. The amplified signal was presented to a pair of ER3A insert earphones (see Ng *et al*., 2013 for technical details). The hearing aid was adjusted to give linear amplification according to each participant's hearing thresholds. In other words, the HINT and SWIR tests were performed in an aided listening condition using the same experimental hearing aid with the same hearing aid signal processing/noise reduction algorithm, which is non-existent in current hearing aids. It is also important to note that whereas the hearing aid signal processing and amplification evaluated in the subjective measures (IOI-HA and SSQ) related to the participants' own hearing aids and settings, the objective measures (HINT and SWIR) related to an experimental hearing aid and settings.

## Results

### IOI-HA and SSQ

The mean responses to the seven items of the IOI-HA and the 10 subscales of the SSQ are shown in Tables 1 and 2 respectively. One of the participants had responses to all items below 2 standard deviations from the mean in IOI-HA and was omitted from all analyses concerning IOI-HA in this paper. The results of the IOI-HA (*n* = 25) and SSQ (*n* = 26) are generally comparable (within 1 SD) to published studies on hearing aid users with more than 6 months of experience (for example, Brännström and Wennerström, 2010; Öberg *et al*., 2007; Gatehouse and Noble, 2004; Köbler *et al*., 2010, respectively).

### Speech recognition test

The mean SNR yielding 84% intelligibility was 2.69 dB SNR (SD = 2.11). No significant correlations were found for any IOI-HA item with SRT. Table 3 shows correlations between the SSQ subscales and measures of SRT. Three out of the four *Qualities of hearing* subscales significantly correlated with the speech reception threshold.

**Table 3:** Correlations between the SSQ and speech reception threshold

SSQ scales	Subscales	Speech reception threshold
Speech hearing	Speech in quiet	−0.49*
	Speech in noise	−0.26
	Speech in speech contexts	−0.38
	Multiple speech-stream processing and switching	−0.28
Spatial hearing	Localization	−0.38*
	Distance and movement	−0.33
Qualities of hearing	Sound quality and naturalness	−0.55**
	Identification of sounds and objects	−0.40*
	Segregation of sounds	−0.51*
	Listening effort	−0.37

**P < 0.01; *P < 0.05.

### SWIR test

The results of the free recall task in the SWIR test in the five background conditions were reported in Ng *et al*. (2013). In the present study, the overall percentage of items correctly recalled in each of the conditions was used in the analyses. No significant correlations were found between the SWIR test and SSQ. The correlations between IOI-HA and the free recall task in the SWIR test are shown in Table 4. Free recall performance in *all* background conditions, with the exception of the 4T/NoP condition, correlated negatively with item 3 of IOI-HA, which measures residual difficulty in adverse listening situations. Correlations with the rest of the items were not statistically significant. Furthermore, factor 2 of IOI-HA, which measures hearing handicap, also correlated with recall performance in the SSN/NoP and 4T/NR.

**Table 4:** Correlations between the IOI-HA and recall performance

IOI-HA items	Quiet	Background noise				Composite score
		Steady-state noise (SSN)		4-talker babble (4T)		
		Without noise reduction (NoP)	Realistic binary masking (NR)	Without noise reduction (NoP)	Realistic binary masking (NR)	
1. Hours of daily use	0.26	0.24	0.20	0.09	0.24	0.31
2. Benefit	0.06	0.10	−0.12	−0.11	−0.01	0.02
3. Residual activity limitations	−0.42*	−0.56*	−0.42*	−0.33	−0.51*	−0.49*
4. Satisfaction	0.18	0.26	0.04	0.05	0.09	0.24
5. Residual participation restriction	−0.36	−0.33	−0.33	−0.21	−0.30	−0.36
6. Impact on others	0.09	−0.12	−0.08	0.01	−0.14	−0.03
7. Quality of life	0.18	0.25	−0.09	0.08	0.12	0.19
Factor 1 (items 1, 2, 4 and 7)	0.34	0.28	0.17	0.21	0.31	0.38
Factor 2 (items 3, 5 and 6)	−0.28	−0.45*	−0.32	−0.20	−0.41*	−0.36

*P < 0.05.

In order to reduce the number of comparisons in the correlation analysis, the results of the memory task in all five background conditions were pooled to give a composite score. The relationship between the free recall composite score and IOI-HA was re-examined (Table 4). Again, only item 3 correlated significantly with the composite score (*P* = 0.013), which reinforces the specificity of the pattern of results.

### Cognitive test battery

Table 5 shows the results of the tests in the cognitive battery. Performance on the reading span test was comparable to those reported in previous studies (Foo *et al*., 2007; Lunner, 2003; Rudner *et al*., 2009; Rönnberg *et al*., 1989, 1998). For the other cognitive tests, the mean response times were within one standard deviation of the mean performance for older adults (mean age = 62 years), reported by Rönnberg (1990).

**Table 5:** Results of the tests in the cognitive battery

	Physical matching	Lexical decision making	Rhyme judgment	Reading span (max. 24)
	Reaction time (ms)		Accuracy (%)	Total recall
Mean	1000.49	979.83	84.61	10.36
SD	234.37	218.15	11.32	3.38

Mean of the reading span test represents average span size in words.

Some of the IOI-HA items and one SSQ subscale significantly correlated with performance on the following cognitive tests: The *Physical matching* test: the reaction time measure significantly correlated with the SSQ subscale *Localization* (*r* = −0.42, *P* = 0.032), indicating that faster processing speed was associated with better self-reported sound localization ability. The *Lexical decision making* test: the reaction time measure significantly correlated with item 1 and factor 1 of the IOI-HA (*r* = −0.43 and −0.40, *P* = 0.032 and 0.048, respectively), indicating that faster lexical processing speed was related to more frequent hearing aid use and better overall hearing aid benefit, yet greater overall reported residual difficulties with hearing aid, and less experienced listening effort. The *Rhyme judgment* test: the accuracy measure (percent correct) significantly correlated with items 1 and 3 of the IOI-HA (*r* = 0.42 and −0.59, *P* = 0.038 and 0.004, respectively), indicating that individuals who used hearing aids more frequently and with better phonological processing reported more remaining difficulties experienced in a challenging listening situation. The correlation with IOI-HA factor 1 was on the verge of significance (*r* = 0.39, *P* = 0.057). The *Reading Span* test: although not statistically significant, a tendency of correlation was observed between the reading span test and item 3 of IOI-HA (*r* = −0.35, *P* = 0.091).

## Discussion

Summarizing the findings, we demonstrated that cognitive measures used in the present study were related to self-reported outcome with own hearing aids and settings. The ability to recall the final words of heard sentences amplified and processed with an experimental hearing aid in all but one of the background conditions was related to reported remaining difficulty in an adverse listening situation (IOI-HA item 3), and performance in two background conditions was related to the overall reported residual difficulty (IOI-HA factor 2). The quality of phonological representations in lexicon, which was measured by the rhyme judgment test, was also related to IOI-HA item 3. These relationships were negative, which mean that individuals with better recall performance reported more remaining difficulties in an adverse listening situation. On the other hand, better overall subjective benefit (IOI-HA factor 1) and more frequent hearing aid usage (IOI-HA item 1) were found to be associated with faster lexical access speed and better quality of phonological representations. It is also worthy of note that the overall recall performance was positively associated, though not significantly, with IOI-HA factor 1. To summarize, cognitive abilities relating to verbal information processing were inversely related to the perceived handicap or residual difficulty experienced in real life, but positively associated with the reported benefits and usage of hearing aid.

### The SWIR test and IOI-HA

Our results suggested that individuals with better recall performance in the SWIR test consistently reported more remaining difficulties in daily situations in which they wanted to hear better. Although we expected that persons having good cognitive abilities would consequently experience less (remaining) problems with hearing aids in daily situations, hence reporting less hearing handicap, our subjective outcome data did not show the expected pattern. One way of explaining the clear and specific pattern of results here is that the self-reported remaining difficulties could actually indicate the relative degree of engagement of explicit processing resources in working memory. In an adverse situation where the hearing aid user wants to hear better, listening is difficult. With the application of appropriate hearing aid amplifications and signal processing algorithms, listening, or even speech communication can be improved. However, hearing aids may partially but not entirely reduce experienced listening difficulties. Thus, the hearing aid user may still encounter or experience remaining difficulties even when listening with a hearing aid, and this is measured by item 3 of the IOI-HA. We assume that the situation described by item 3 is an adverse listening situation, such as when the target speech is degraded or presented in the presence of competing noise. Mattys *et al*. (2012) summarized that listening in an adverse situation may lead to poor matching of segmental and lexical representations and consumption of additional cognitive resources. Thus, having high cognitive capacity would result in better speech perception and less remaining difficulties in such a situation. Conversely, however, more explicit processing would have to be engaged to achieve language understanding when the quality of input speech signal becomes suboptimal, as hypothesized by Rönnberg (2003) and Rönnberg *et al*. (2008) in the working memory model for Ease of Language Understanding. Listening in adverse situations requires extra processing of speech in working memory, which is mentally effortful. Therefore, the responses made to item 3 may relate to the listening situations where explicit processing is involved. Previous studies have suggested that the experienced processing demand and engagement of explicit processing are associated with perceived effort in listening (Grady, 2012; Hällgren *et al*., 2005; Koelewijn *et al*., 2012; Pichora-Fuller, 2003; Rudner *et al*., 2012). We argue that responses to item 3 of IOI-HA, which focuses on the remaining difficulty experienced by the hearing aid user when using hearing aid(s), reflect the amount of explicit processing experienced or, in other words, how effortful it was to listen in challenging situations.

High performers on the free recall task (SWIR) have better cognitive spare capacity (Mishra *et al*., in press) than low performers. In other words, they have more remaining cognitive resources to remember the words that they successfully identified. The finding of more experienced remaining hearing difficulty for persons with better cognitive spare capacity is mirrored in findings relating to quality of phonological representations. Performance on the rhyme judgment task was also negatively correlated with item 3 of IOI-HA, suggesting that listeners with better quality of phonological representations in the lexicon experienced more remaining hearing difficulty. In adverse listening situations, the process of matching the lexical information of input signals to the contents of long-term memory storage becomes effortful and explicit. The individuals who have better cognitive spare capacity and thus more working memory resources at their disposal and/or better quality phonological representations in the lexicon would be better able to engage in explicit kinds of processing in adverse listening situations than the individuals who have poorer quality phonological representations and limited cognitive spare capacity. Therefore, the higher performers on the free recall task and the rhyme judgment task are more likely to find it to be more effortful to listen in such situations in real life. That is why they reported more remaining difficulty than low performers even though they actually had the ability to perform better.

By the same argument, those who performed worse in the free recall task had relatively limited cognitive resources and hence experienced less effortful explicit processing. Therefore, they reported less residual difficulty. Koelewijn *et al*. (2012) also showed a result pattern where people with good cognitive ability, who performed better than those with limited ability in a speech recognition task, actually consumed more effort (measured in terms of pupil dilation) in order to achieve high performance in the task. Grady (2012) hypothesized that brain activity increases when cognitive load increases. In particular, older adults who have better memory and cognition are able to activate their brains more intensively than those who have higher risk of impaired memory performance as task demands increase. In other words, individuals with better cognitive ability seem to be more capable to utilize explicit resources when the task is increasingly demanding. The results of these studies are consistent with the directionality of the correlation pattern observed in the present study.

Besides the hypothesis related to cognitive resources discussed above, there are other possible explanations that could have accounted for more remaining listening difficulties being reported by hearing aid users with better recall performance. For instance, persons with better cognitive skills, who have greater benefit from hearing aids (Akeroyd, 2008), may also have greater expectations of successful listening than persons with poorer cognitive skills. Although positive expectations are associated with better hearing aid outcome in general (for example, Cox and Alexander, 2000; Saunders *et al*., 2009), over-expectation may also result in disappointment. Thus, hearing aid users who have higher expectations may tend to report more difficulties, especially in challenging listening situations where hearing aid benefit is relatively limited, than those who have realistic expectations. Indeed, Saunders *et al*. (2004) reported that better speech recognition performance in noise is correlated with underestimation of hearing ability. Thus, self-reported outcome may vary with factors such as expectation and self-perception, and these factors could have contributed to the observed pattern of results in the present study.

### Cognitive test battery and self-reported outcomes

Better quality of phonological representations and faster lexical access speed were associated with more frequent reported daily use and also better success with hearing aids. Hearing aid amplification with advanced signal processing can greatly improve speech perception and understanding, but listening remains difficult in complex or adverse listening situations. Moreover, the advanced signal processing in hearing aids may distort speech signals. Good quality phonological representations and rapid lexical access are crucial in decoding speech input under suboptimal conditions, for example, when the speech input is distorted or presented in noise (Rönnberg, 2003; Rönnberg *et al*., 2008). Therefore, individuals with better such cognitive abilities may find hearing aids more beneficial (especially in adverse situations) than those with limited abilities, and thus tend to wear their hearing aids more frequently. The present study establishes the link between cognitive abilities and self-reported hearing aid use and overall success, which extends the findings of previous studies showing a link between cognitive skills and the ability to distinguish between (Lunner, 2003) and perceived benefit of (Gatehouse *et al*., 2006) different hearing aid signal processing algorithms.

The quality of phonological representation, though correlated with better overall hearing aid success, was also associated with more remaining hearing difficulty in challenging listening situations and handicap. These two findings are not necessarily contradictory although one may expect that for those who report more hearing aid benefit/success would report less remaining hearing difficulty. On the one hand, we know that individuals with better cognitive abilities engage more explicit processing in a suboptimal listening situation (Koelewijn *et al*., 2012) and may hence report more remaining difficulty and handicap; on the other hand, with remedial and explicit processing, these individuals could achieve better speech understanding and better hearing aid use and thus report greater overall benefit/success. Thus, we argue that more reported hearing difficulty but greater reported hearing aid success are complementary and not contradictory to each other.

There was a tendency towards a correlation (*r* = −0.35, *P* = 0.09) between item 3 of the IOI-HA and the reading span test. Item 3 was found to be significantly correlated with the recall performance of the SWIR test. For the same group of participants, the recall performance of the SWIR test was significantly correlated with the reading span test (Ng *et al*. 2013). Thus, a significant relationship between item 3 and the reading span test was expected. The reading span test is visually based and is developed in a way so that working memory is taxed explicitly (the cognitive resources for recalling either the first or the final word of sentences in a list is competed with the resources for judging the semantic content of the sentences). For the SWIR test (repeating the final word of sentences and recalling all the repeated final words in a list), working memory is also taxed because the nature of the two tests are similar. However, the speech stimuli in the SWIR test were presented auditorily in noise (stationary noise and speech babble). Listening and remembering speech in a noisy background in the SWIR test probably resembles more closely than the reading span test the adverse listening situation described by item 3, where difficulties still remain in the presence of hearing aid amplification. Therefore, item 3 of the IOI-HA was significantly correlated with the recall performance of the SWIR test but not with the reading span test. Nevertheless, the directionality of the correlation was the same as for the SWIR test, which is reasonable.

In the other self-report outcome measure used in the present study (SSQ), there were correlations between speech reception threshold and five subscales (Speech in quiet in the Speech hearing domain, Localization in the Spatial hearing domain, and Sound quality and naturalness, Identification of sounds and objects and Segregation of sounds in the Qualities of hearing domain) and only one signification correlation was found between general processing speed and the *Localization* subscale in the Spatial hearing domain. Unlike the IOI-HA, the SSQ is more focused on various listening abilities, including discrimination of voice, hearing of sounds and the ability to identify speech in different situations (ranging from easy to challenging situations), which have been found to be associated with speech recognition thresholds in previous studies. No question pinpoints the overall difficulties or even remaining difficulties in listening in adverse conditions, in which working memory is taxed substantially. Thus, the SSQ was found to be less effective than IOI-HA in revealing the relationship between self-report and cognitive measures in the present study.

Since speech tests alone cannot always satisfactorily predict self-reported benefits (Taylor, 2007), further studies should explore the possibility of using cognitive behavioral measures, such as the cognitive test battery used in the present study, to predict real-world hearing aid use and benefits. Recall performance in most of the background conditions test was found to be related to item 3 of the IOI-HA. The SWIR test, which involved perceiving and processing of speech stimuli, resembles listening in challenging real-life situations. Therefore, behavioral tests using a similar paradigm could be employed to predict aided listening performance in real life. An experimental hearing aid was used in the behavioral tests. Although the amplification was individually prescribed, the participants might not have been acclimatized to the new settings, which could have partially altered the results of the study. Therefore, it will be preferable to have the participants' own hearing aids to perform future behavioral tests.

## Conclusions

This study demonstrated a relationship between cognitive measures and residual difficulty reported by hearing aid users. The results showed that reported residual difficulty with hearing aids may relate to experienced explicit processing in difficult listening conditions, such that individuals with better cognitive capacity tended to report more remaining difficulty in challenging listening situations. Cognitive abilities related to verbal information processing are also associated with self-reported hearing aid use and overall success, such that individuals with better cognitive abilities reported better hearing aid use and overall success. The possibility of using cognitive measures to predict hearing aid outcome in real life should be explored in future.
